# Diagnostic challenges of long COVID in children: a survey of pediatric health care providers’ preferences and practices

**DOI:** 10.3389/fped.2024.1484941

**Published:** 2024-12-23

**Authors:** Vivian Y. Liu, Madeleine Godfrey, Matthew Dunn, Robert Fowler, Lauren Guthrie, David Dredge, Scott Holmes, Alicia M. Johnston, Tregony Simoneau, Alessio Fasano, Dawn Ericson, Lael M. Yonker

**Affiliations:** ^1^ Department of Pediatrics, Massachusetts General Hospital, Boston, MA, United States; ^2^ Department of Pediatrics, Harvard Medical School, Boston, MA, United States; ^3^ Department of Pediatrics, Pulmonary Division, Massachusetts General Hospital, Boston, MA, United States; ^4^ Division of Pulmonary Medicine, Boston Children’s Hospital, Boston, MA, United States; ^5^ Department of Neurology, Massachusetts General Hospital, Boston, MA, United States; ^6^ Department of Anesthesia, Critical Care and Pain Medicine, Boston Children’s Hospital, Boston, MA, United States; ^7^ Division of Infectious Diseases, Boston Children’s Hospital, Boston, MA, United States; ^8^ Department of Pediatrics, Gastroenterology Division, Massachusetts General Hospital, Boston, MA, United States

**Keywords:** pediatric, SARS-CoV-2, COVID-19, post-viral illness, post-acute sequalae of COVID, PASC, long COVID

## Abstract

**Introduction:**

Given the challenges in diagnosing children with long COVID, we sought to explore diagnostic practices and preferences among clinicians.

**Methods:**

A ten-question survey assessed pediatric providers’ clinical decision making for identifying and evaluating long COVID in children. Of the 120 survey respondents, 84 (70%) were physicians, 31 (26%) nurse practitioners, and 5 (4%) physician assistants.

**Results:**

The most common categories of symptoms identified as raising suspicion for long COVID in children included cardiopulmonary symptoms, selected by 119 (99%) of pediatric providers, and neurocognitive symptoms, selected by 118 (98%) of providers. However, there was more ambiguity on the primary feature of long COVID, with providers selecting a range of key symptoms. Of all physical exam findings, postural orthostatic tachycardia, was most suggestive of long COVID [identified by 49 (41%) of pediatric providers], whereas one-third of providers reported no specific identifiable exam finding.

**Discussion:**

Pediatric providers report variable decision making in the clinical evaluation of long COVID, with patient demographics and clinical factors impacting whether a diagnosis of long COVID is considered. This variation in diagnosing pediatric long COVID reflects ambiguity in the definition of long COVID in children and the absence of clinical guidelines to support providers in the identification of disease and treatment. This study highlights an area of need for future clinical advances in pediatric long COVID.

## 1 Introduction

While advances have been made to diagnose, treat, and reduce risk of severe illness with acute COVID-19 (1), the understanding of post-acute sequalae of COVID (PASC) lags, especially in pediatric populations where the impact of SARS-CoV-2 infection has been challenged since the outset of the pandemic (2, 3). The indolent but disruptive form of PASC, termed long COVID, is associated with persistent and/or recurrent symptoms months after SARS-CoV-2 infection. The current definition of long COVID includes the development of any over 200 possible symptoms occurring over 4 weeks after recovery from the acute infection with SARS-CoV-2, in the absence of an alternative diagnosis (4). While the more severe version of PASC, multisystem inflammatory syndrome in children (MIS-C) (5), was rapidly recognized as a COVID-related clinical entity, long COVID in children has been slower to become conceptualized.

One of the reasons why long COVID is harder to identify in children may be because of the broad and ambiguous presentation of long COVID in children (6), as well as a lack of standardized diagnostics or management guidelines for pediatricians. In adults, there have been advances in recognizing long COVID. There are iterations of clinical guidelines to help providers evaluate for long COVID in adults (7), although there are no established objective diagnostic tools. In comparison, there is a paucity of guidance for pediatric providers. The lack of guidelines in pediatrics fuels questions of legitimacy of long COVID in children, delays diagnosis, and hinders understanding of the natural progression of disease and advances in therapeutic strategies.

Here, we surveyed pediatric providers to determine practitioners’ preferences for evaluating long COVID in children. The goal of this study was to determine which clinical symptoms and factors elicit consideration of long COVID and gain insight into providers’ diagnostic strategies. This data can inform future efforts in refining the clinical guidelines of this condition in pediatrics.

## 2 Materials and methods

This study was approved by Mass General Brigham (IRB Protocol 2023P003129). All data obtained from participants who completed the voluntary, self-administered questionnaire was confidential and stored in a HIPAA-compliant, secure REDCap database.

### 2.1 Participants

Between November 2023 and January 2024, a digital survey was sent to a convenience sample of pediatric providers. In exchange for participant time and input, participants were given the option to enter a raffle for gift cards upon completion of the survey. Inclusion criteria required that participants hold a MD/DO, NP, and/or PA degree, and that they specialize in pediatrics, pediatric subspecialties, emergency medicine, and/or family medicine. These criteria were selected to ensure the relevance of pediatric long COVID to the clinical practice of our target survey participants.

### 2.2 Survey

We developed a digital survey to better understand clinical practices when evaluating for long COVID in children. The survey questions were reviewed by physician researchers with experience treating pediatric long COVID. The survey included 10 questions divided into 2 sections: (1) Signs and symptoms of long COVID in children, and (2) Diagnostic approach to evaluating symptoms of long COVID.

Survey respondents were asked about symptom duration and timing of presentation relative to acute infection in patients across all pediatric age groups, as well as risk factors such as sex, age, race/ethnicity, socioeconomic status, and immunization status. In the second section, respondents were prompted to select tests to order, if any, and queried whether they have referred patients for subspecialty evaluation. The questionnaire was designed to take 5–10 min to complete. The blank survey can be found in Supplementary Figure S1.

### 2.3 Analysis

Data collected on RedCAP and descriptive analyses are reported. Fisher's Exact test was implemented to assess contingency between two groups using GraphPad Prism (Version 10.2.3). *P*-values < 0.05 were considered significant.

## 3 Results

**Figure 1:**
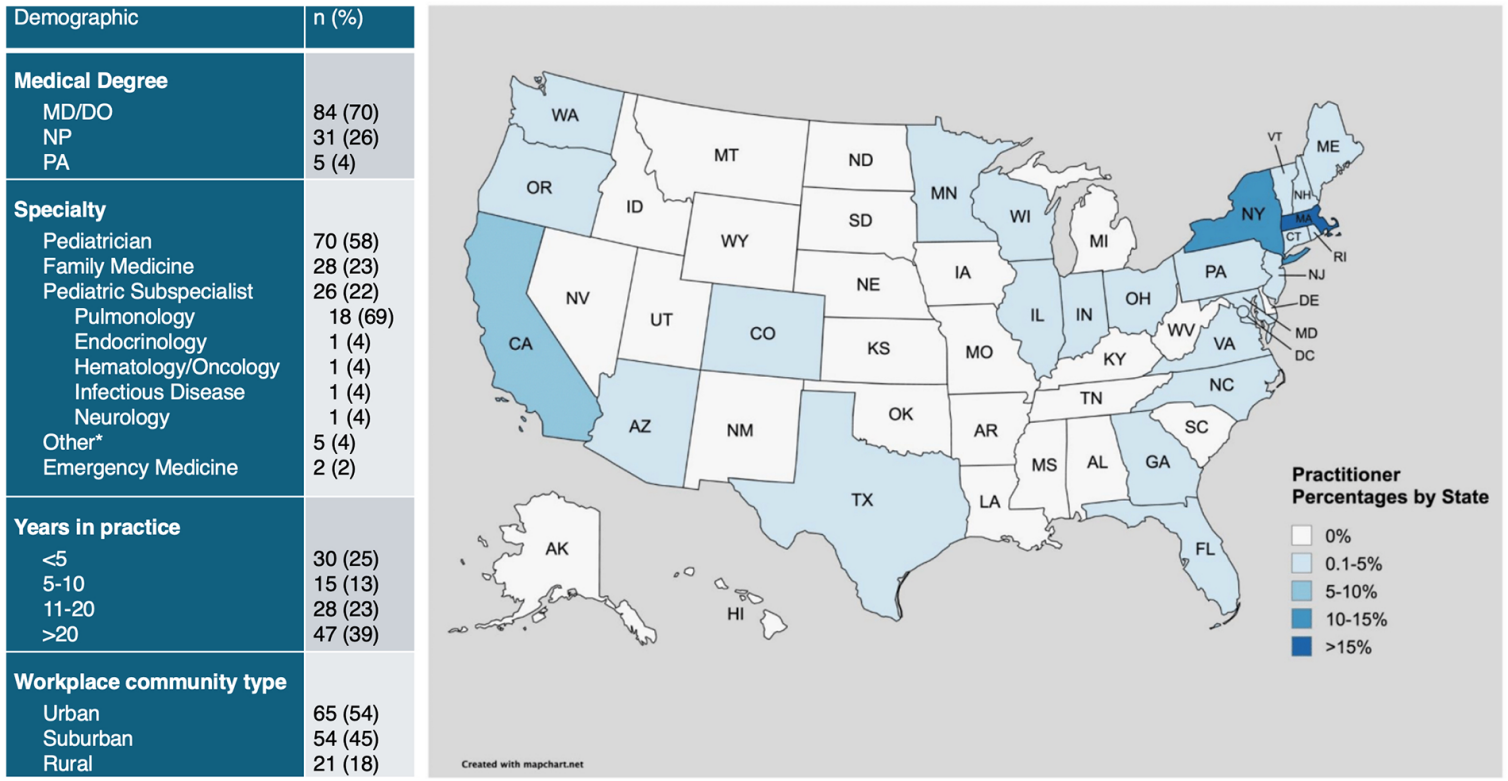
Provider demographics and geographic distribution. Demographic information of providers including medical degree, specialty, years in practice, and workplace community type. Geographic distribution of providers is visualized on the map. *Other specialties include 1 sleep medicine, 1 psychiatry, 1 primary care, and 2 unspecified specialties. Created with mapchart.net.

A total of 120 participants completed the survey (Figure 1). Respondents were comprised of 84 (70%) physicians, 31 (26%) nurse practitioners, and 5 (4%) physician assistants. Responder specialty included 70 (58%) primary care pediatricians, 28 (23%) family medicine, 26 (22%) pediatric subspecialists, and 2 (2%) emergency medicine providers. While a range of subspecialists responded to the survey, many of the respondents (*n* = 18; 69%) were pediatric pulmonologists, potentially reflecting referral patterns in practice. Many respondents had been in practice for over 20 years (*n* = 47, 39%), while thirty (25%) have been in practice <5 years, representing a wide range of clinical experience among respondents. Geographically, 96% of respondents practice in 26 states across the United States. Two respondents practice in Mexico, one in Canada, and one in Argentina. In terms of community setting, 65 (54%) work in urban areas, 54 (45%) in suburban regions, and 21 (18%) in rural communities; 18 (15%) report working in more than one community setting.

### 3.1 Cardiopulmonary and neurocognitive symptoms are key triggers for consideration of long COVID

Pediatric providers were asked to select new-onset signs and symptoms that would lead them to consider a diagnosis of long COVID in children (Table 1). The most common categories of symptoms selected were cardiopulmonary and neurocognitive; 119 (99%) of providers reported cardiopulmonary symptoms and 118 (98%) of respondents reported neurocognitive symptoms as key features raising concern for long COVID. Cardiopulmonary symptoms most selected were intolerance of activity (91%), shortness of breath (75%), and post-exertional malaise (73%). Chronic cough (67%) and patient reports of palpitations or tachycardia (57%) were also common symptoms raising clinical suspicion for long COVID. From the neurocognitive category, many providers selected brain fog (91%), increased fatigue (84%), and persistent sensory complaints (63%). Anxiety, depression, excessive headache, and dizziness were also commonly reported as symptoms concerning for long COVID. New onset hyperactivity was identified as a symptom concerning for long COVID by only 10% of respondents.

**Table 1:** New onset symptoms concerning for long COVID categorized by class of symptoms most to least likely selected by pediatric providers.

Symptom	*n* (%)
Cardio/pulmonary	119 (99)
Intolerance of usual activities or exercise	109 (91)
Shortness of breath/difficulty breathing	90 (75)
Post-exertional malaise	87 (73)
Chronic cough	80 (67)
Palpitations or tachycardia	68 (57)
Chest pain	47 (39)
Neurocognitive	117 (98)
Brain fog/memory problems	109 (91)
Fatigue or tiredness	101 (84)
Persistent sensory complaints	76 (63)
Anxiety	58 (48)
Depression	53 (44)
Excess headache	53 (44)
Dizziness	51 (43)
Hyperactivity	12 (10)
Other	103 (86)
Changes in taste or smell	86 (72)
Sleep disturbance	59 (49)
Other	1 (1)
No symptoms make me concerned for Long COVID	1 (1)
Musculoskeletal	85 (71)
Myalgia	80 (67)
Arthralgia	56 (47)
Abnormal movements	26 (22)
Endocrine	73 (61)
Menstrual changes	51 (43)
Hair loss	42 (35)
Changes in urinary habits	18 (15)
Increased thirst	15 (13)
Gastrointestinal	66 (55)
Abdominal pain	54 (45)
Nausea/vomiting	36 (30)
Diarrhea	26 (22)
Constipation	16 (13)

Musculoskeletal symptoms including myalgias (67%) and arthralgias (47%) were identified as symptoms related to long COVID in children. Gastrointestinal symptoms, such as abdominal pain (45%) and nausea (30%), and endocrine symptoms, such as menstrual changes (43%) and hair loss (35%), were also frequently selected symptoms suggestive of long COVID.

**Figure 2:**
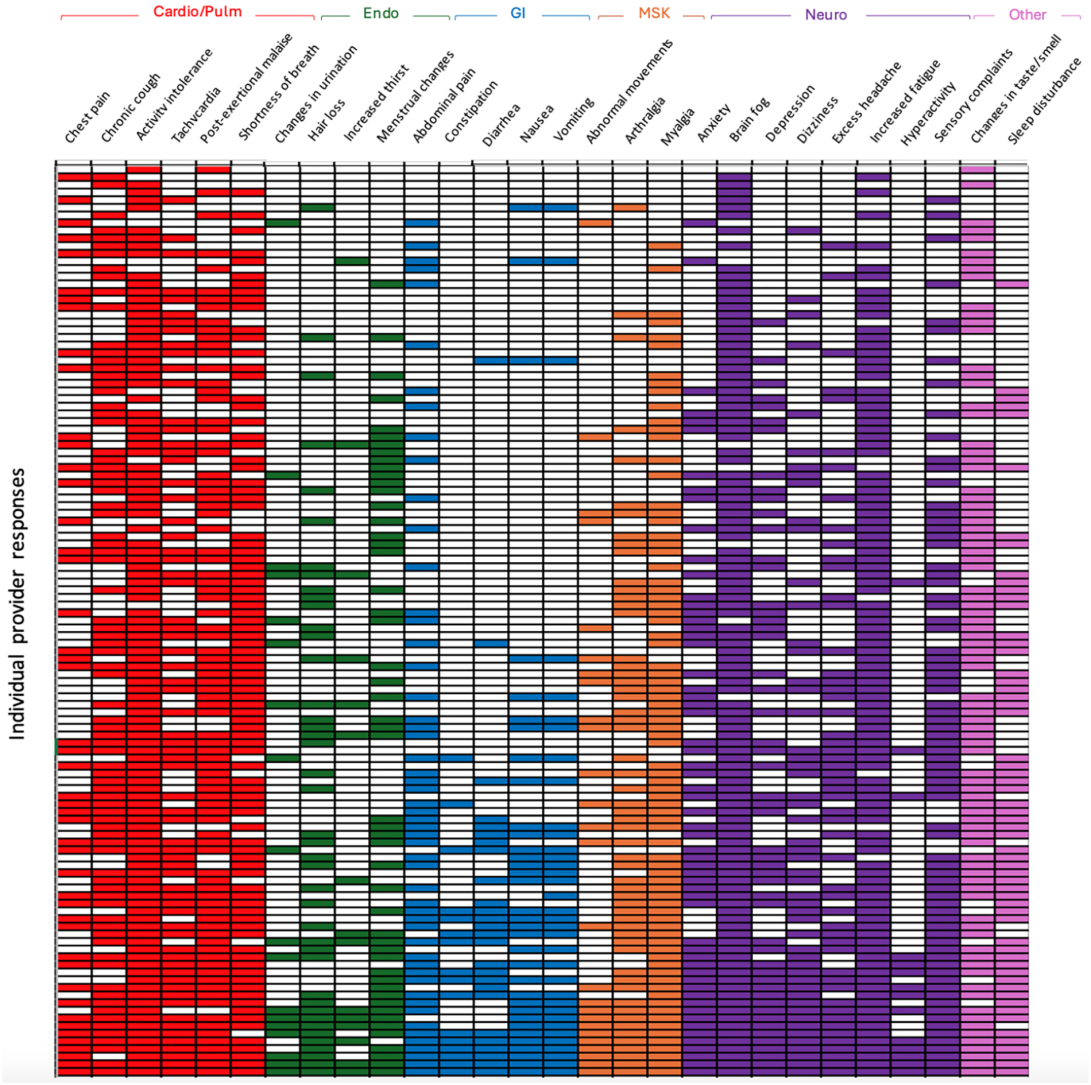
Selected symptoms concerning for long COVID by each individual provider. Each row corresponds to an individual provider's selection of new onset symptoms concerning for long COVID. Rows are organized from top to bottom as least to most selected total number of symptoms. (Cardio/Pulm, cardiopulmonary; Endo, endocrine; GI, gastrointestinal; MSK, musculoskeletal; Neuro, neurocognitive).

Most pediatric providers (*n* = 119, 99%) selected more than one organ system involvement in long COVID, with 86 (72%) selecting at least 10 symptoms. This highlights provider preference for assessing multi-organ involvement when evaluating for long COVID. Individual provider selected responses are shown in Figure 2.

**Figure 3:**
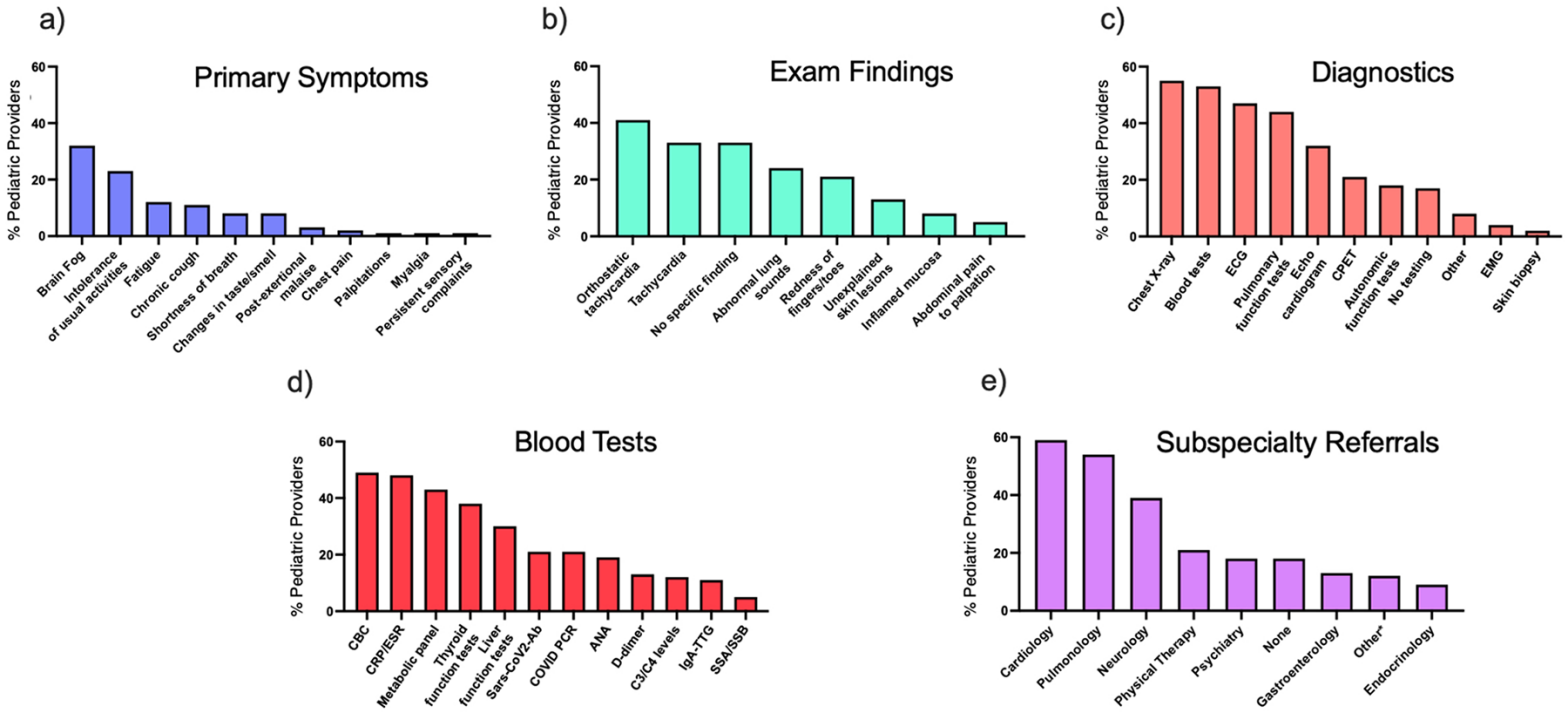
Long COVID clinical presentation and provider diagnostic preferences. Percent of providers selecting **(a)** key primary symptoms and **(b)** exam findings concerning for long COVID. **(c)** Diagnostic tests ordered by pediatric providers including **(d)** specific blood tests. **(e)** Percent of providers referring patients to subspecialists if there is clinical concern for long COVID. (ECG, electrocardiogram; CPET, cardiopulmonary exercise testing; EMG, electromyogram; CBC, complete blood count; CRP/ESR, C-reactive protein/elevated sedimentation rate; ANA, anti-nuclear antibody; IgA-TTG, immunoglobulin A-tissue transglutaminase; SSA/SSB, anti-Ro/anti-La antibodies).

In order to prioritize symptoms and identify key features that raise clinical suspicion for long COVID in children, we asked pediatric providers to identify a single symptom that is most concerning for long COVID. Brain fog/memory problems (*n* = 38, 32%) and intolerance of usual activities or exercise (*n* = 28, 23%) were indicated as key features most concerning for long COVID (Figure 3a).

### 3.2 Clinical exam findings and diagnostic tests offer limited guidance in identifying children with long COVID

Pediatric providers were prompted to select exam findings that would lead them to consider long COVID in children if clinical suspicion was present. While no clinical exam findings were diagnostic of long COVID, the most frequently selected finding suggestive of long COVID was postural orthostatic tachycardia, which was selected by *n* = 49 (41%) of pediatric providers. Other frequently selected symptoms included tachycardia (at rest) (33%), abnormal lung sounds (24%), unexplained skin lesions (13%), inflamed mucosa (8%) and abdominal pain elicited by palpation (5%). One-third of respondents selected that no specific findings triggered a diagnosis of long COVID (Figure 3b).

To understand current clinical evaluation patterns, providers were asked what clinical tests they would order when evaluating for long COVID in children. The majority of pediatric providers (*n* = 66, 55%) would order a chest x-ray and 64 (53%) would order laboratory blood tests. Electrocardiogram (ECG), pulmonary function tests, cardiopulmonary exercise testing (CPET), and autonomic function tests were also often considered by pediatric providers. Seventeen percent would opt for no testing at all (Figure 3c**)**. For providers who reported they would order laboratory tests, many would order a complete blood count (CBC) (59%), c-reactive protein/erythrocyte sedimentation rate (CRP/ESR) (57%), basic metabolic panel (BMP) (52%), and thyroid function tests (46%). Liver tests, SARS-CoV-2 antibody or viral testing by polymerase chain reaction (PCR), anti-nuclear antibody (ANA), d-dimer, C3/4 levels, immunoglobulin A tissue transglutaminase (IgA-TTG), and anti-Ro/anti-La (SSA/SSB) were also considered by pediatric providers (Figure 3d).

### 3.3 Providers are likely to refer patients to cardiologists, pulmonologist, and neurologists for evaluation of long COVID

Providers were asked whether they would refer patients to a subspecialist for further evaluation of long COVID. The vast majority (*n* = 98, 82%) of responding providers would refer their patients for subspecialty evaluations. The specialty most referred to was cardiology (59%), followed by pulmonology (54%) and neurology (39%). Other reported referrals included gastroenterology (13%) and endocrinology (9%). 18% would not refer their patients to a subspecialist. 18% chose an “Other” specialty, which included infectious disease (6%), rheumatology (3%), and specialty long COVID or other multidisciplinary clinics (3%) (Figure 3e).

### 3.4 Clinical context, including time since infection, patient demographics, and provider experience with long COVID impacts likelihood of diagnosis

**Figure 4:**
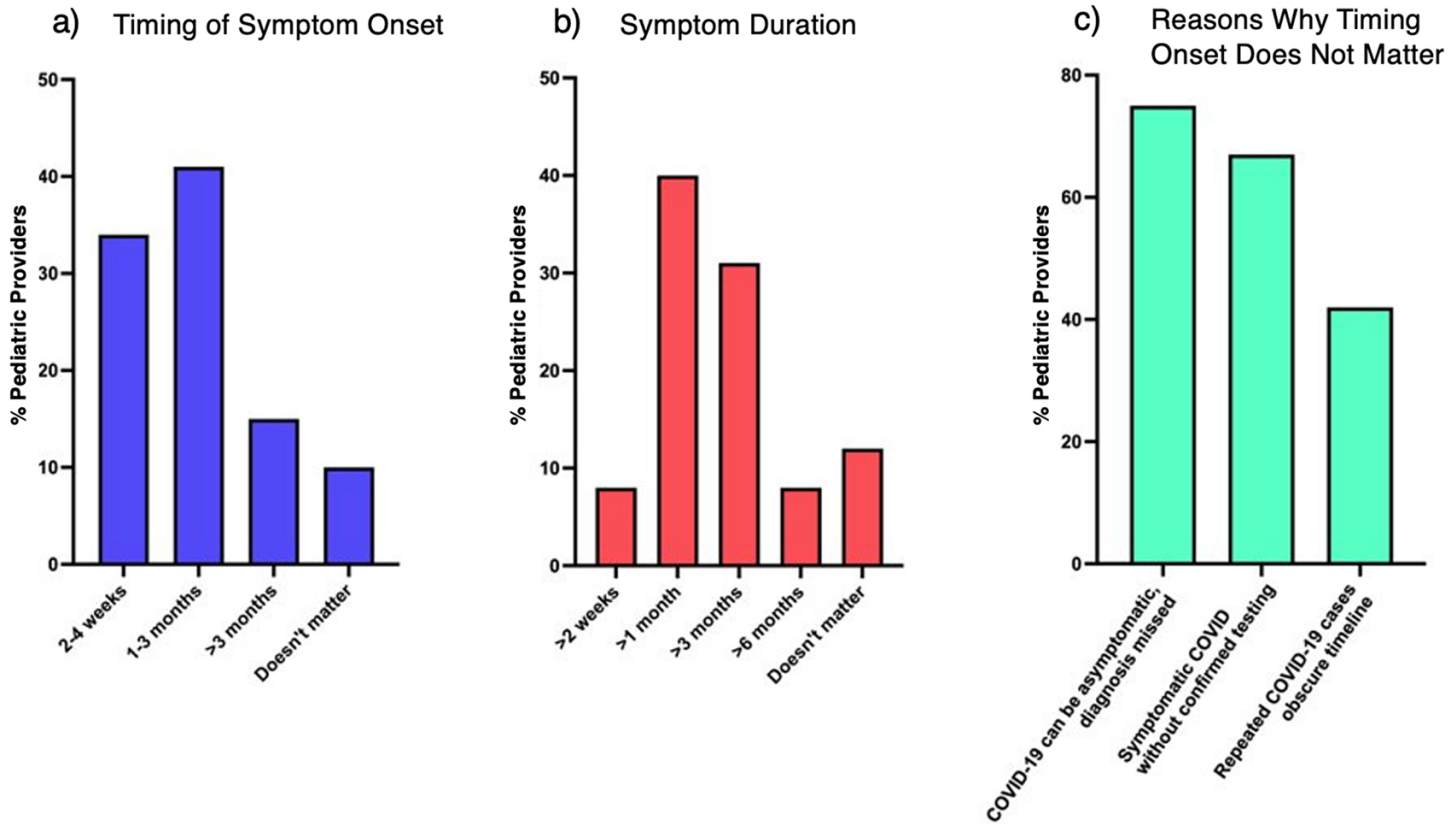
Provider understanding of long COVID symptom timeline. Percent of providers who would consider a diagnosis of long COVID based on **(a)** timing of symptom onset after acute infection and **(b)** symptom duration. **(c)** Reasons why providers chose timing of symptom onset does not matter.

When asked about timing of presentation, most (*n* = 66, 55%) would be more likely to consider long COVID if symptom onset was at least 1 month post infection. Of that group, 15% would have a higher suspicion for long COVID if symptoms were >3 months post-acute infection. Other pediatric providers (*n* = 41, 34%) would consider long COVID if symptoms were 2–4 weeks post-acute infection. Twelve percent reported that timing of symptom onset does not matter when considering a diagnosis of long COVID (Figure 4a).

When asked about duration of symptoms, most (*n* = 106, 88%) reported duration of symptoms does matter when assessing for long COVID. Forty-eight (40%) would have suspicion for long COVID if symptoms lasted >1 month. Thirty-six (30%) would consider long COVID if symptoms lasted >3 months. Other pediatric providers (*n* = 9, 8%) selected symptom duration 2–4 weeks long and another 9 providers (8%) selected >6 months. Twelve percent reported symptom duration does not affect consideration of long COVID in pediatric patients (Figure 4b).

Out of those who selected that symptom timeline does not affect their likelihood of diagnosing long COVID, 9 (75%) said the possibility of asymptomatic COVID was a factor behind that selection. Other factors include the possibility of symptomatic COVID without confirmed testing (*n* = 8, 67%) and prevalence of patients having repeated cases of COVID (*n* = 5, 42%), which makes a clear timeline between acute and post-acute symptoms difficult to determine (Figure 4c).

**Figure 5:**
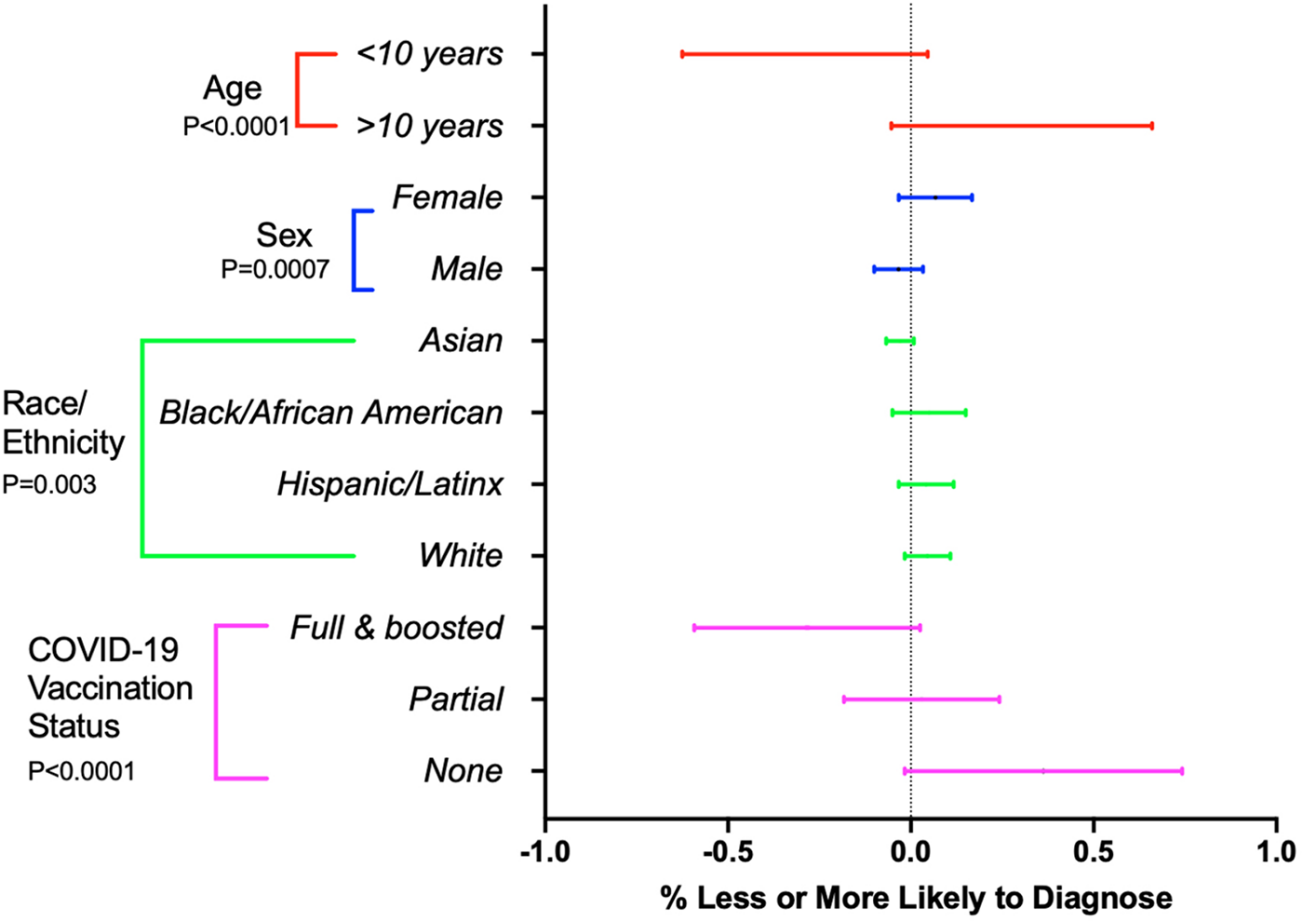
Influence of patient demographics on provider diagnosis of long COVID. Forest plot representing percent of providers who are more (1.0) or less likely (−1.0) to consider a long COVID diagnosis based on age, sex, race/ethnicity, and COVID-19 vaccination status. Horizontal bars indicate 95% confidence intervals. *P*-values < 0.05 are considered significant.

We then sought to ascertain whether patient demographics impacted the likelihood of a patient being diagnosed with long COVID. Survey participants were asked to select risk factors that would make them less likely, neutral, or more likely to diagnose long COVID in their pediatric patients (Figure 5). Age was a significant factor with 78% of respondents reporting they would be less likely to consider a diagnosis of long COVID in children under 10 years of age as compared to children over 10 years of age (Fisher's exact test, *p* < 0.0001). Females were significantly more likely to be considered for long COVID (Fisher's exact test, *p* = 0.007). Race and ethnicity also impacted provider likelihood for considering long COVID, with African Americans being more likely to be considered for long COVID and Asian patients being the least likely (Fisher's exact test, *p* = 0.003). COVID-19 vaccination status showed significant influence over the likelihood of providers diagnosing long COVID in children, with pediatric providers more likely to diagnose long COVID in unvaccinated patients as compared to patients who received their full COVID-19 vaccination series (Fisher's exact test, *p* < 0.0001).

**Figure 6:**
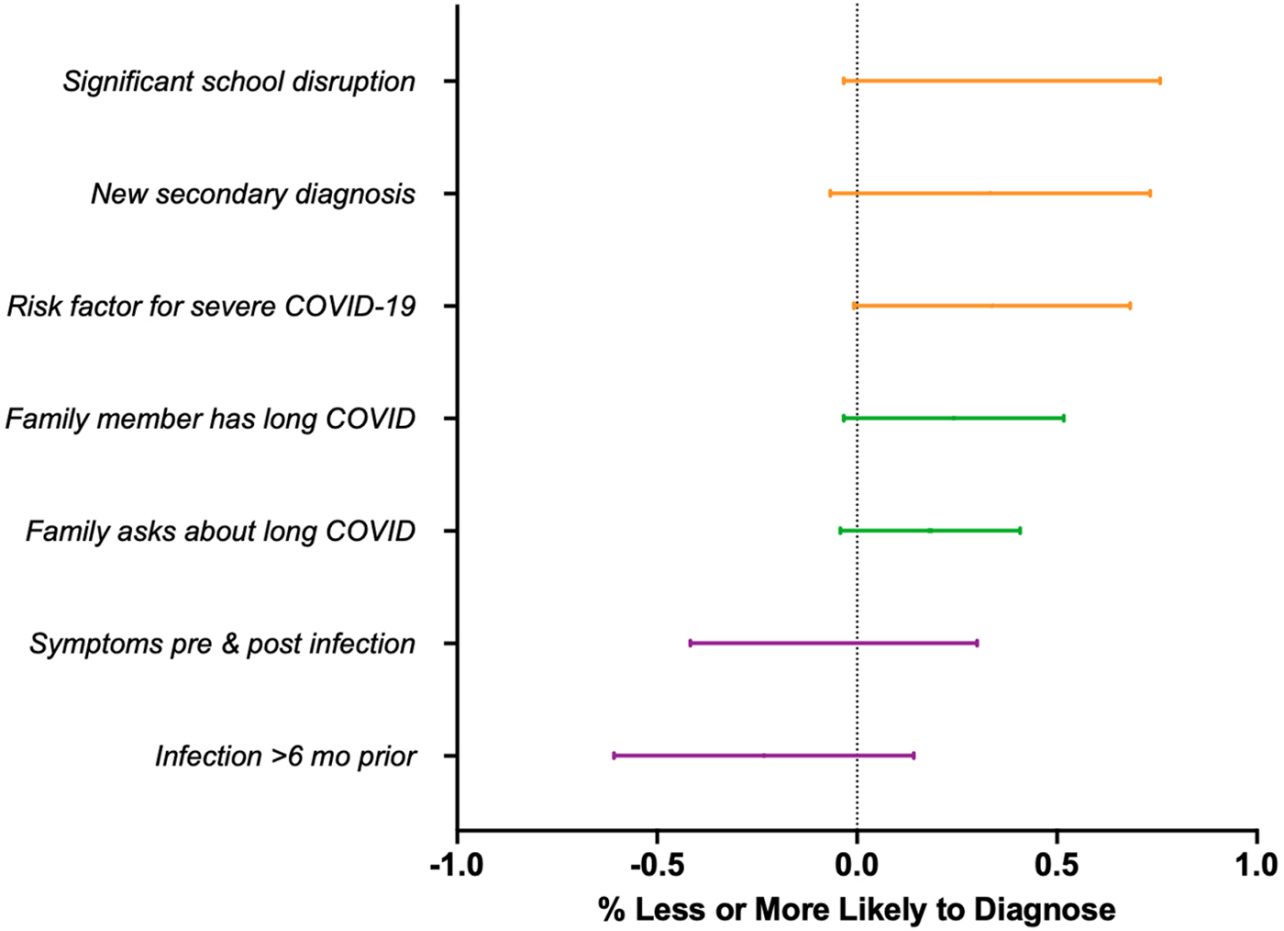
Influence of other clinical considerations on provider diagnosis of long COVID. Forest plot representing percent of providers who are more (1.0) or less likely (−1.0) to consider a long COVID diagnosis based on other relevant clinical features.

Other features that made providers more likely to diagnose long COVID included significant school disruption (76%), new secondary diagnoses such as a new autoimmune disease or blood clots (73%), and comorbid conditions such as obesity and diabetes (68%) leading to increased risk for severe COVID-19. Family history of long COVID, and family-initiated conversations about the possibility of long COVID also increased the likelihood of considering a diagnosis of long COVID in a pediatric patient. Features that would decrease practitioners’ likelihood of diagnosing long COVID were acute SARS-COV-2 infection >6 months prior to presentation with full recovery (61%) and full immunization against COVID-19 prior to symptom onset (60%) (Figure 6).

To assess prevalence of long COVID among provider patient panels, pediatric providers were asked to estimate how many children in their panel have been diagnosed with long COVID. While many pediatric providers (*n* = 65, 54%) report not having any patients in their panel diagnosed with long COVID, 42 (35%) have 1–5 pediatric patients with long COVID on their patient panel, and 13 (11%) have greater than 6 patients. A minority of pediatric providers (*n* = 15, 13%) reported that they have patients whom they suspected had long COVID but did not discuss the condition with the patients/families. Main reported reasons for not discussing long COVID included limited clinical treatments and lack of a diagnostic test to confirm the diagnosis. To assess general acceptance and familiarity of long COVID as a diagnosis in adults, we asked whether providers knew any adults with long COVID; two-thirds of pediatric providers (*n* = 80, 67%) knew an adult with a diagnosis of long COVID (Table 2).

**Table 2:** Number of long COVID diagnoses in provider panels and potential barriers for diagnosing pediatric long COVID.

Response	*n* (%)
How many children in your patient panel have been diagnosed with long COVID?	
0	65 (54%)
1–5	42 (35%)
>6	13 (11%)
Do you have patients whom you suspect have long COVID but haven't discussed this as a possibility with the patient/family?	
Yes	15 (13)
No	105 (88)
Reasoning for not discussing long COVID with patients suspected to have the condition:	
Clinical treatments are limited and symptom-based	13 (87)
No diagnostic test to confirm long COVID	8 (53)
No pediatric long COVID referral centers are available	5 (33)
Long COVID is similar to other post-viral syndromes	3 (20)
Diagnosis would increase anxiety in patient/family	2 (13)
I'm not convinced that long COVID is real	1 (7)
Do you know any adults with long COVID?	
Yes	80 (67)
No	40 (33)

## 4 Discussion

While over 65 million adults report symptoms of long COVID based on US Centers for Disease Control and Prevention surveillance data (8), prevalence data in pediatrics is variable (2). Without clear diagnostic criteria or testing, it is challenging to identify and diagnose long COVID especially in children. Here, we sought to gain insight into common pediatric practitioner practices and clinical decision making when evaluating children presenting with possible long COVID. The study demonstrates marked variability in providers’ threshold for considering long COVID and diagnostic work-up.

Existing clinical guidelines are inadequate for diagnosing pediatric long COVID in the clinic. While over 200 symptoms of long COVID have been identified (4, 9) and many pediatric practitioners associate a range of symptoms across multiple organ systems, as demonstrated in this study, more refined clinical criteria and guidance may be helpful for focusing assessments in clinical practice. Symptomology based on age groups may also be helpful as the presentation of long COVID can vary between school-aged children and older adolescents (10). In this study, neurocognitive and cardiopulmonary symptoms are key symptoms that trigger a diagnosis of long COVID across all age groups. Many studies report neurologic and cognitive complaints as primary presenting symptoms of long COVID, including mood swings, trouble remembering or concentrating, and fatigue (11, 12). Studies also document persistent sensory complaints in long COVID (13), similar to neuropathy triggered by other viral respiratory infections (14). Cardiopulmonary symptoms such as shortness of breath and chest tightness predominate in long COVID as well (15). As reflected in this study, many providers are already prioritizing and grouping symptoms.

While defining symptomatic criteria is essential for diagnosis, objective data needs to be assessed. Our study revealed pediatric providers view postural orthostatic tachycardia as suggestive of long COVID, but no exam findings were felt to be indicative of long COVID. One out of three providers reported no specific findings concerning for long COVID on exam. Similarly, clinical work-up was broad and nonspecific, including chest x-ray and labs such as CBC, CRP/ESR, BMP, and thyroid function tests. The utility of these tests lies in helping to potentially rule out other diagnoses but are not diagnostic for long COVID. Targeted diagnostic tests are urgently needed. One recent study looked at lung ultrasound as an evaluative tool, but initial results reported it was not suitable for assessment and follow-up in pediatric long COVID (16). Continued research into diagnostic pathways sequencing utility of tools and referrals is necessary before focusing on therapeutics.

Referral to specialty services may be needed for certain diagnostic tests. Our results demonstrate referral preferences to cardiology, pulmonology and neurology, which align with the primary presenting cardiopulmonary and neurocognitive symptoms of patients. The role of specialists has not yet been defined, despite their potential important contributions to comprehensive testing and evaluation. For example, the field of cardiology is vital to the multidisciplinary care of long COVID patients. Cardiopulmonary exercise testing and echocardiograph studies could help characterize long-term autonomic cardiac symptoms in affected patients (17, 18). Recognizing and defining the roles of specialists would be helpful.

Interestingly, many demographic considerations impact the likelihood of being diagnosed with long COVID. As we have seen since the beginning of the pandemic, both physiologic (19) and socioeconomic features (20, 21) have impacted which populations of individuals are at highest risk for SARS-CoV-2 related diseases. Here, we show there are demographic features that affect the likelihood of being diagnosed with long COVID. Specifically, older children, females, and families with other members diagnosed with long COVID are more likely to be diagnosed with long COVID, especially if the family initiated the conversation. Conversely, children younger than 10 years of age, males, and individuals of Asian descent are less likely to be diagnosed with long COVID. While objective diagnostic criteria for long COVID remain elusive, it is unclear if this reflects the true incidence of long COVID in different demographic groups or whether this reflects potential barriers to diagnosis.

Growing evidence suggests that persistent reservoirs of SARS-CoV-2 may be linked to long COVID symptoms (22); persistent SARS-CoV-2 RNA is an indicator of these reservoirs, and has been identified in patients with long COVID through tissue biopsies (23) and RT-PCR conducted on stool, urine, or plasma samples (24). Moreover, multiple studies analyzing plasma from patients with long COVID have found detectable levels of SARS-CoV-2 proteins over one-year post-acute infection (25, 26). These proteins may enter the bloodstream through the intestinal mucosal barrier due to intestinal reservoirs of SARS-CoV-2 and subsequent gut dysbiosis (27). Studies have looked at potential biomarkers of long COVID including decreased serum cortisol (28), cytokines profiles (25, 29), microclots (30), or shifts in non-conventional monocytes or T cell populations (28). Other routinely available lab values have been tested in large adult cohorts but were not found to be clinically useful or meaningful in the diagnosis of long COVID (31). While persistent viral reservoirs are associated with long COVID, there are no recognized laboratory biomarkers that can support the symptom-based diagnosis of long COVID. Investigation into novel biomarkers is needed.

Defining clinical and diagnostic criteria for children with signs of long COVID is essential. Affected patients may quietly endure prolonged physical and emotional symptoms for years. Children with long COVID report concern that others may judge them negatively due to their diagnosis of long COVID or may not believe long COVID is a real disease (32), and therefore may be less likely to voice their symptoms to providers and caregivers. While the pandemic itself significantly impacted children's quality of life, with educational and social disruptions, the symptomatic burden of long COVID adds additional psychological distress and biologic pathophysiology in affected children. Symptoms including brain fog, decreased concentration, and sensory changes lead to decreased engagement in their usual daily activities. While long COVID has been reported to have an economic impact in adults of over 3.7 trillion dollars, based on decreased spending, reduced earnings, and increased medical utilization costs (33), the lasting cost of long COVID on children cannot yet be realized. These children and adolescents need to be recognized so that providers are intentional about asking questions regarding mental health, scheduling close follow-ups, and providing multidisciplinary support as needed.

While this study offers insight into clinical practice and provider preferences when evaluating long COVID in children, our respondents represent a convenience sample, both geographically and by provider specialty. However, we captured a range of practitioners across multiple clinical settings with a range of clinical experience. We found that many providers have provided clinical care for no or few patients with long COVID, which means their clinical preferences in this survey study may be swayed by external factors rather than personal clinical experience and practice. Some providers may have seen patients with long COVID without recognizing it due to limited guidance in evaluating pediatric long COVID as highlighted by the results of this study. Also, patients may not be routinely seeking medical care for symptoms of long COVID or families are directly pursuing care at specialized long COVID clinics. Regardless, our results show variability in the clinical practice, which must be better understood to improve outcomes in children with long COVID symptoms.

## 5 Conclusion

Long COVID not only affects adults but also children and adolescents. Overall, our study demonstrates that pediatric providers share understanding in the symptomatic presentation of long COVID in children and adolescents, but practice is variable and further objective evaluation, diagnostic tools, and referral indications are undefined. While certain tests can help rule out other diagnoses, further research into focused diagnostic tools such as biomarkers will help facilitate diagnosis and guide treatment recommendations. Clinical advances are urgently needed to increase recognition and diagnosis of long COVID when pediatric patients report concerning symptoms.

## Data Availability

The original contributions presented in the study are included in the article/Supplementary Material, further inquiries can be directed to the corresponding author.
