# Supplementary material for: Diagnostic challenges of long COVID in children: a survey of pediatric health care providers’ preferences and practices
Source: Front Pediatr. 2024 Dec 23;12:1484941. doi: 10.3389/fped.2024.1484941 (PMC11700732; doi:10.3389/fped.2024.1484941)
Supplement: Supplementary file 1 [file Image1.pdf]

**REDCap:**
**We are trying to understand practitioner's preferences in evaluating children for Long COVID. Thank you for helping us better understand how Long COVID is evaluated in the pediatric setting.**

**Signs and Symptoms of Long COVID in Children**:

1. Which of these **new-onset symptoms** would lead you to consider Long COVID (select all):

Cardio/Pulmonary
- Chest pain
- Chronic cough
- Intolerance of usual activities or exercise
- Palpitations or tachycardia
- Post-exertional malaise
- Shortness of breath/difficulty breathing

Endocrine:
- Changes in urinary habits
- Hair loss
- Increased thirst
- Menstrual changes

Gastrointestinal:
- abdominal pain
- constipation
- diarrhea
- Nausea
- Vomiting

Muscular/Skeletal
- Abnormal movements
- Arthralgia
- Myalgia

Neurologic/Cognitive
- Anxiety
- Brain fog/difficulty thinking, concentrating, forgetfulness, or memory problems
- Depression
- Dizziness
- Excess headache
- Hyperactivity
- Increased fatigue or tiredness
- Persistent sensory complaints (tingling, pain, itch, odd sensations, distress from normal contacts)

Other
- Changes in taste or smell
- Sleep disturbance

- Other, please specify

No symptoms make me concerned for Long COVID

2. Of those symptoms selected, what is the **primary symptom** that makes you most likely to consider Long COVID?:

3. What **exam findings** lead you to consider Long COVID in children (select all)?
    a. Abdominal pain elicited by palpation
    b. Abnormal lung sounds
    c. Inflamed mucosa
    d. Postural orthostasis
    e. POTS (excess rise in heart rate upon standing)
    f. Rapid heartbeat (tachycardia)
    g. Redness in fingers and/or toes
    h. Unexplained skin lesions/rash
    i. Other, please specify
    j. No specific exam findings make me concerned for Long COVID

4. When considering a diagnosis of Long COVID, does it matter **when the child had acute infection**? (select 1)
    a. Child had COVID-19 2-4 weeks prior to symptom onset
    b. Child had COVID-19 1-3 months prior to symptom onset
    c. Child had COVID-19 >3 months prior to symptom onset
    d. Time since COVID-19 doesn't matter
        i. Why doesn't timing matter? (select all that apply)
            1. COVID-19 can be asymptomatic in children and diagnosis could have been missed
            2. Most children/adolescents have had COVID-19 multiple times so timing is less relevant
            3. The patient may not have tested for COVID-19 when they were symptomatic

5. Does **symptom duration** matter for diagnosing Long COVID: (select 1)
    a. Duration of symptoms doesn't matter
    b. Symptoms last >2 weeks
    c. Symptoms last >1 month
    d. Symptoms last >3 months
    e. Symptoms last >6 months

6. Are you more or less likely to diagnose Long COVID in the following scenarios: (all less/neutral/more)
    a. Age

        i. Age <5 years
        ii. Age 5-9 years
        iii. Age 10-14 years
        iv. Age 15+ years
        v. Adult (>21 years)
    b. Sex:
        i. Patient identifies as female
        ii. Patient identifies as male
    c. Race/Ethnicity:
        i. Asian
        ii. Black/African American
        iii. Hispanic/Latinx
        iv. White
    d. Socioeconomic status:
        i. Patient lives in wealthy area
        ii. Patient lives in socioeconomically disadvantaged area
    e. Immunization status:
        i. Patient was fully immunized and boosted against COVID-19 prior to symptom onset
        ii. Patient has partial COVID-19 vaccination but was not up to date with COVID-19 vaccines/boosters prior to symptom onset
        iii. Patient did not receive any COVID-19 immunizations
    f. Additional considerations:
        i. Patient had SARS-CoV-2 infection > 6 months prior and recovered without complication
        ii. Family asks about Long COVID as a possible diagnosis
        iii. Other family members have Long COVID
        iv. Patient has obesity, diabetes, or other risk factor for severe COVID-19
        v. Patient has new secondary diagnosis (ie: new autoimmune disease, new blood clots)
        vi. Symptoms result in significant school disruption
        vii. Symptoms were present prior to COVID-19 but now have worsened or returned

**Diagnosis:**

1. When evaluating for Long COVID in children, what tests do you order: (select all)
    a. Autonomic function testing
    b. Cardiopulmonary exercise testing (CPET)
    c. CXR
    d. ECG
    e. Echo
    f. Lab tests
        i. ANA

ii. Basic metabolic panel
iii. CBC
iv. COVID pcr
v. CRP/ESR
vi. C3, C4 complement levels
vii. D-dimer
viii. IgA-TTG for celiac
ix. LFTs
x. SARS-CoV-2 antibodies
xi. SSA/SSB for Sjogren's
xii. Thyroid function tests

g. Nerve conduction testing EMG for muscle and large-fiber nerve conditions
h. Pulmonary function tests
i. Skin biopsy testing for small-fiber neuropathy
j. Other, please specify
k. No testing

2. When evaluating for Long COVID in children, have you referred for subspeciality evaluations? (select all that apply)
    a. Cardiology
    b. Endocrinology
    c. Gastroenterology
    d. Neurology
    e. Physical Therapy
    f. Psychiatry
    g. Pulmonary
    h. Other, please specify
    i. None

3. How many children in your patient panel have been diagnosed with Long COVID?
    a. Fill in number

4. Do you have patients who you suspect have Long COVID but haven't discussed this as a possibility with the patient/family
    a. No
    b. Yes
        i. What is/are the reason(s) for not discussing Long COVID as a diagnosis? (select all)
            1. Clinical treatments are limited; I address symptoms instead.
            2. I'm not convinced that Long COVID is real.
            3. Long COVID is similar to other post-viral syndromes.

4. No diagnostic test is available to confirm Long COVID
5. No Pediatric Long COVID referral centers are available.
6. Would increase anxiety in patient/family.

Do you know any adults with Long COVID? Y/N

**Some questions about you:**

1. What is your medical degree (select all that apply):
    a. MD/DO
    b. NP
    c. PA

2. What is your field of specialty (select all that apply):
    ii. Pediatrician
    iii. Pediatric subspecialist
        1. Allergy/immunology
        2. Cardiology
        3. Endocrinology
        4. Gastroenterology
        5. Hematology/Oncology
        6. Infectious diseases
        7. Neurology
        8. Pulmonology
        9. Pediatric surgical subspecialty
        10. Other, please specify
    iv. Emergency Medicine
    v. Family Medicine
    c. How many years have you been in practice? (select one)
        i. <5
        ii. 5-10
        iii. 11-20
        iv. >20
    d. In which state do you work? (If you work outside the US, write country) (fill in)
    e. Select community type (select all)?
        i. Urban
        ii. Suburban
        iii. Rural

**Comments/Concerns: _____**
